# Transcription Factor Expression in Sinonasal Neuroendocrine Neoplasms and Olfactory Neuroblastoma (ONB): Hyams’ Grades 1–3 ONBs Expand the Spectrum of SATB2 and GATA3-Positive Neoplasms

**DOI:** 10.1007/s12022-022-09715-3

**Published:** 2022-05-06

**Authors:** Silvia Uccella, Carla Facco, Anna Maria Chiaravalli, Fabiana Pettenon, Stefano La Rosa, Mario Turri-Zanoni, Paolo Castelnuovo, Michele Cerati, Fausto Sessa

**Affiliations:** 1 Unit of Pathology, Dept. of Medicine and Surgery, University of Insubria, via O. Rossi 9, 21100 Varese, Italy; 2 Dept. of Pathology, ASST Dei Sette Laghi, Varese, Italy; 3 Unit of Otorhinolaryngology, Department of Biotechnology and Life Sciences, University of Insubria, Varese, Italy

**Keywords:** Olfactory neuroblastoma, Sinonasal neuroendocrine carcinoma, Amphicrine carcinoma, MiNEN, SATB2, GATA3, CDX2

## Abstract

Sinonasal neuroendocrine neoplasms (SN-NENs) are rare and mostly include neuroendocrine carcinoma (NEC), whereas neuroendocrine tumor (NET) is exceptional in this site. Olfactory neuroblastoma (ONB) is a malignant neuroectodermal neoplasm arising in the nasal cavity. Albeit crucial for correct patients’ management, the distinction of high grade ONB from NEC is challenging and requires additional diagnostic markers. The transcription factor SATB2 has been recently introduced in routine diagnostics as an immunohistochemical marker of distal intestine differentiation. No specific data are available about SATB2 and GATA3 expression in SN-NENs. GATA3, SATB2, and, for comparison, CDX2 expression were investigated in a series of epithelial and non-epithelial SN-NENs. We collected 26 cases of ONB and 7 cases of epithelial SN-NENs diagnosed and treated in our Institution. ONBs were graded according to Hyams’ system and epithelial NENs were reclassified into 5 NECs, 1 MiNEN, and 1 amphicrine carcinoma. Immunohistochemistry was performed using standard automated protocols. Hyams’ grades 1–3 ONBs stained diffusely and intensely for SATB2, whereas grade 4 ONBs and NECs were globally negative. The non-neuroendocrine component of MiNEN and the amphicrine carcinoma were strongly positive. GATA3 was heterogeneously and unpredictably expressed in Hyams’ grades 1–3 ONBs, whereas grade 4 ONBs and NECs were completely negative. CDX2 was negative in all cases. Our study identifies, for the first time, SATB2 and GATA3 expression as features of Hyams’ grades 1–3 ONBs, expands the spectrum of SATB2 and GATA3-positive neoplasms, and suggests that Hyams’ grade 4 ONBs are not only clinically but also biologically different from low graded ONBs.

## Introduction

Sinonasal neuroendocrine neoplasms (SN-NENs) are rare and mostly include neuroendocrine carcinomas (NECs) of the large and small cell subtype [1]. A subset of NECs is mixed with non-neuroendocrine carcinomas and can be considered mixed neuroendocrine/non-neuroendocrine neoplasms (MiNENs), as it happens in other digestive and extra-digestive sites [2–4]. The differential diagnosis of SN-NEC encompasses several high-grade neoplasms, including lymphoma, rhabdomyosarcoma, melanoma, Ewing sarcoma, sinonasal undifferentiated carcinoma, and high grade olfactory neuroblastoma (ONB), as well as molecularly defined high-grade carcinomas such as NUT carcinoma and SWI/SNF-deficient carcinomas [1, 5]. The proper diagnosis, typing, and classification of sinonasal neoplasms has important clinical consequences and impacts on patients’ management. In particular, the distinction between NEC and ONB, which strongly expresses general neuroendocrine markers, as well as the histological grading (Hyams’ grade) of ONB, is crucial in predicting patients’ outcome [6, 7]. If morphological features are the corner stone for the differential diagnosis between NECs and low grade ONBs, the use of immunohistochemical markers is mandatory for distinguishing NECs from high-grade ONBs (particularly, Hyams’ grade 4 ONBs). The diagnosis of ONB relies on diffuse positive staining for general neuroendocrine markers (chromogranin A and synaptophysin) in neoplastic cells, together with S100-positivity in sustentacular cells (which are very few in grade 4 ONB) and global negativity for cytokeratins (also if a subset of cases may be focally positive). NECs do not present S100-positive cells and express cytokeratins together with general neuroendocrine markers [2, 6, 7]. Indeed, there are cases in which the distinction of a NEC from a high-grade ONB is challenging and there is need for additional markers helping the diagnosis [8].

Special AT-rich sequence-binding protein 2 (SATB2) is a DNA binding protein acting as a transcription factor and as a docking site for several chromatin remodeling enzymes. In the embryogenesis, it is involved in skeletal development and in palate formation. In the adult, it is highly expressed in brain neurons and in epithelial cells of large intestine, appendix, and renal convoluted tubules and moderately expressed in non-germinal center cell of secondary lymphoid organs, in the testis, in the epididymis, and in osteoblasts [9]. The availability of anti-SATB2 antibodies for diagnostic immunohistochemistry is a recent acquisition in the immunohistochemical armory of the pathologist. Among their main applications, the most employed in surgical pathology are the identification of the primary site of metastatic adenocarcinomas of unknown origin, and the differential diagnosis between primary and metastatic mucinous adenocarcinomas of the ovary, being SATB2 a sensitive and specific marker for appendiceal and colonic differentiation [10, 11]. In neuroendocrine pathology, this marker has been shown to be selectively expressed in gastrointestinal well-differentiated neuroendocrine tumors (NETs), particularly in appendiceal and rectosigmoid NETs, but not in pancreatic or pulmonary NETs [12, 13], showing a distinct expression pattern compared to CDX2, another intestine-specific transcription factor [14]. Recently, SATB2 expression has also been found in middle ear NETs [15]. In turn, among neuroendocrine carcinomas (NECs), a significant proportion of Merkel cell carcinomas (> 75%) express SATB2, which is positive in no more than a third of NECs of other sites [12, 16, 17]. Non-epithelial neuroendocrine neoplasms (NENs), including pheochromocytoma and paraganglioma, have been reported to poorly express SATB2 [12], whereas they are consistently GATA 3-positive [18].

In this study, we aim to investigate, for the first time, the immunohistochemical expression of SATB2, GATA3, and CDX2 in a series of well-characterized SN-NENs and to explore their possible role in diagnostic daily practice of endocrine and head and neck pathologists.

## Materials and Methods

Twenty-six cases of ONB diagnosed between 2005 and 2020, two of which were lymph node metastases, were randomly extracted from the archives of the Pathology Service of our Institution that is a referral center for sinonasal surgery, in which a total of 62 cases of ONB were excised and diagnosed in the same period. The frequency distribution of Hyams’ grades in the sample analyzed was comparable to that of the whole series of cases in our Institution. For comparison, 7 randomly pulled cases of neuroendocrine neoplasms (NENs) of the nasal cavity were included in the study. For each case, the availability of histological sections for the histopathological review and for additional immunohistochemistry was verified. Clinical data (age, sex, stage of disease, follow up data) were available for all cases.

The histopathological review of all cases was performed by expert head and neck pathologist (CF), neuropathologist (MC), and endocrine pathologist (SU). The minimum immunohistochemical panel used for the diagnosis included CK AE1/AE3, CK8, synaptophysin, chromogranin A, INSM1, S100, NUT, SMARCA4, SMARCB1 (INI1), myogenin, MYOD1, CD45, and CD99. The diagnosis of NEC required a suggestive morphology, positivity for cytokeratins (at least CK8) and at least two general neuroendocrine markers, retention of SMARCA4 and SMARCB1 (INI1), and global negativity for S100, NUT, myogenin, MYOD1, CD45, and CD99. The diagnosis of low grade ONB was established in presence of a consistent morphology, expression of neuroendocrine markers and presence of S100-positive sustentacular cells. High-grade ONB was diagnosed in presence of a neuroendocrine marker-expressing, cytokeratin-negative neoplasm with retention of SMARCA4 and SMARCB1 (INI1), and global negativity for S100, NUT, myogenin, MYOD1, CD45, and CD99. Once the histopathological diagnosis was established, Hyams grade was applied for ONB and NENs were classified according the IARC/WHO proposal for a common classification framework [19].

Immunohistochemistry for SATB2, GATA3, and CDX2 was performed on 3-µm-thick whole sections of representative formalin-fixed and paraffin-embedded samples of each tumor and automatically processed on Ventana BenchMark Ultra immunostainer. Specific antibodies anti-SATB2 (Clone EP281, Roche Diagnostics), anti-GATA3 (Clone L50-823, Roche Diagnostics), and anti-CDX2 (clone EPR2764Y, Roche Diagnostics) were applied after heat induced antigen retrieval according to standardized protocols.

The immunostains were evaluated semi-quantitatively, and both the extent and intensity of the stain were recorded for each case. In detail, the percentage of positive over the total neoplastic cells was evaluated, with 10% intervals, whereas the intensity of the immunostain was defined as faint (1 +), moderate (2 +), or strong (1–3 +). In addition, a multiplicative score was calculated, by multiplying the intensity score by the absolute value of cell percentage.

## Results

After the histopathological review, the 26 ONBs were subdivided, according to Hyams’ grade, into 2 grade 1, 13 grade 2, 8 grade 3, and 3 grade 4. The two lymph node metastases of ONB were Hyams’ grade 2 and grade 4, respectively. As for NENs, 6 cases were confirmed and classified in 3 small cell NECs (SCNEC), 2 large cell NECs (LCNEC), and 1 MiNEN (large cell NEC and intestinal type adenocarcinoma, ITAC). The remaining case was reclassified as an amphicrine carcinoma (neoplastic cells had coexistent features of neuroendocrine and intestinal type adenocarcinoma differentiation).

**Fig. 1:**
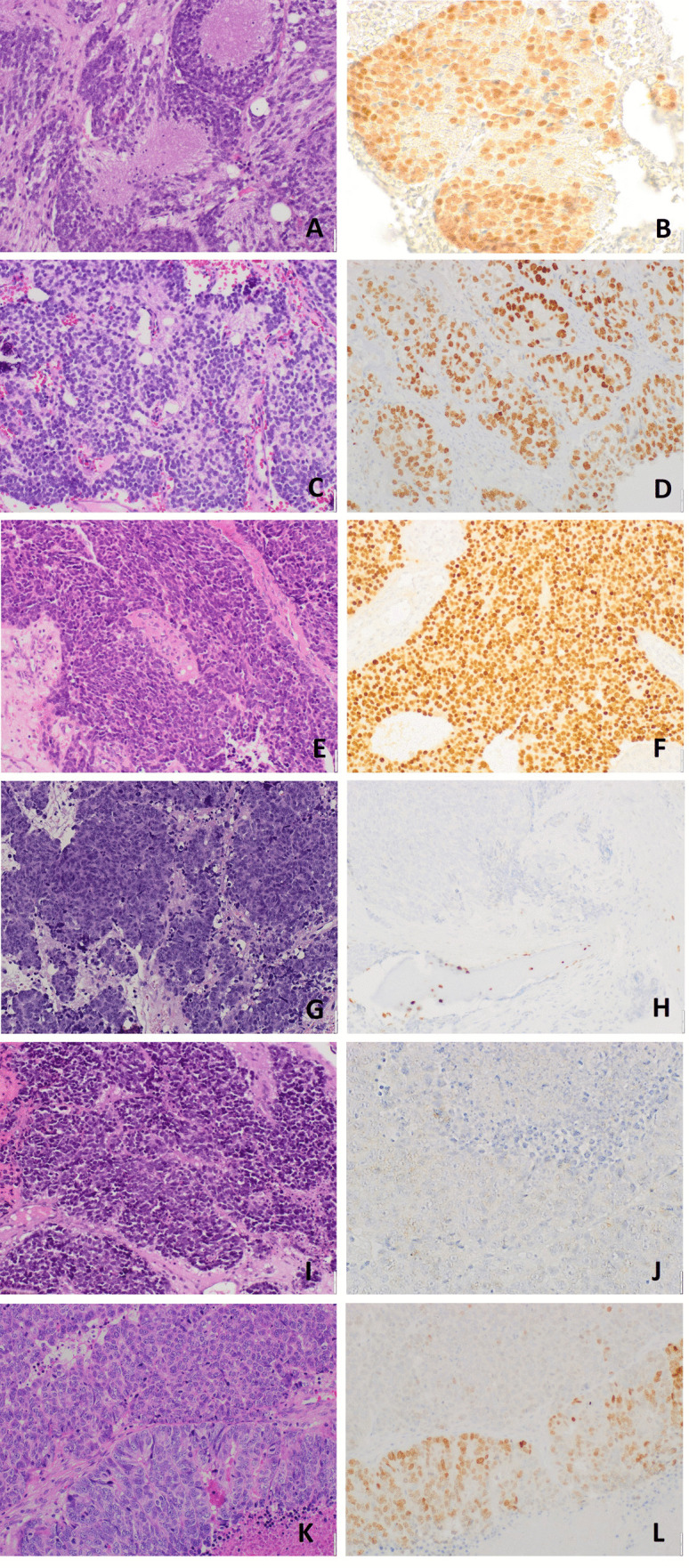
Immunostains for SATB2 in sinonasal NENs. SATB2 was intensely and diffusely expressed in Hyams’ grade 1 (**A**-**B**), grade 2 (**C**-**D**), and grade 3 (**E**–**F**) ONBs, whereas it was mostly negative in Hyams’ grade 4 ONBs (**G**-**H**, positive internal control: osteoblasts) and NECs (**I**-**J**). The NEC component of the MiNEN (**K**) was negative, as well, whereas the ITAC component showed an expected intense and diffuse positivity (**L**)

**Fig. 2:**
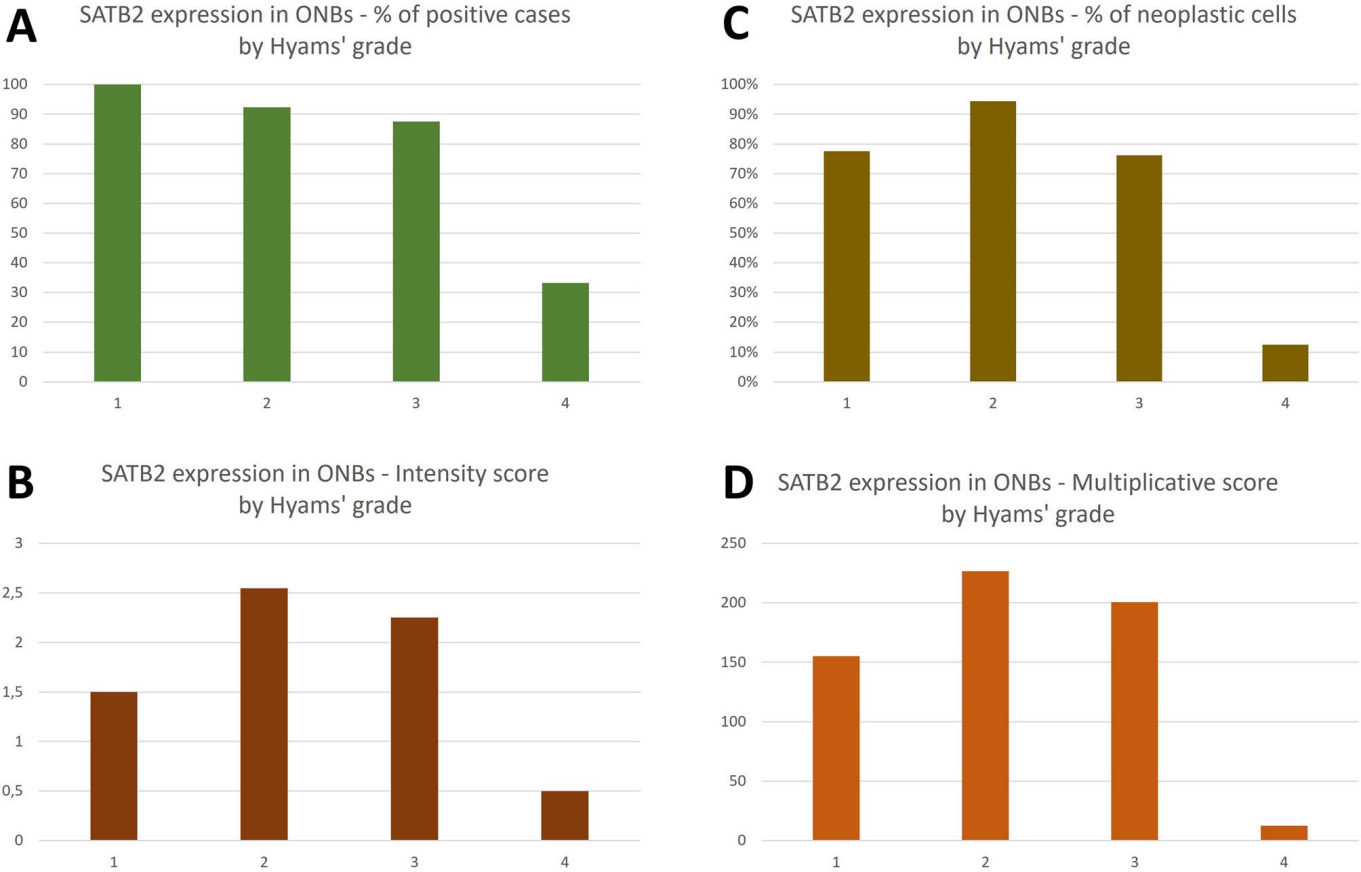
Summary of immunohistochemical analysis of SATB2 expression in ONBs by Hyams’ grade, in terms of percentage of positive cases (**A**), percentage of positive neoplastic cells (**B**), intensity score (**C**), and multiplicative score (**D**)

The results of the immunohistochemical analysis are detailed in Table 1 and illustrated in Fig. 1. SATB2 expression was significantly different in ONBs compared to NENs. Overall, ONBs showed SATB2 expression in 22 out of 26 cases (84.6%), whereas all NENs, including the 5 NECs and the neuroendocrine component of the MiNEN, were negative. The ITAC component of the MiNEN and the amphicrine carcinoma were intensely positive for SATB2 in all neoplastic cells. Intriguingly, when ONBs were considered according to Hyams’ grade, we found significant variations in term of both the intensity of the immunostain and of the percentage of positive neoplastic cells. First, 2 out of 3 grade 4 ONB were negative for SATB2, with the remaining one showing only faint positivity in a low fraction of cells (25%). In contrast, among grades 1, 2, and 3 ONBs, only two cases were negative (1 grade 2 and 1 grade 3), whereas all the others showed moderate to intense immunostaining in 50% or more of neoplastic cells (Fig. 2).

**Table 1:** Immunohistochemical expression in STAB2, GATA3, and CDX2 in sinonasal neuroendocrine neoplasms

**N**	**Tumor diagnosis**	**Grade/Subtype**	**SATB2**			**GATA3**			**CDX2**
			%	Intensity score	Moltiplicative score	%	Intensity score	Moltiplicative score	%
ONBs									
1	ONB	Hyams 1	70	2 +	140	15	2 +	30	0
2	ONB	Hyams 1	85	3 +	170	3	3 +	9	0
3	ONB	Hyams 2	95	2 +	190	0	-	-	0
4	ONB	Hyams 2	100	3 +	300	0	-	-	0
5	ONB	Hyams 2	100	3 +	300	0	-	-	0
6	ONB	Hyams 2	100	2 +	200	1	2 +	2	0
7	ONB	Hyams 2	100	3 +	300	2	1 +	2	0
8	ONB	Hyams 2	100	2 +	200	2	1 +	2	0
9	ONB	Hyams 2	0	-	-	0	-	-	0
10	ONB	Hyams 2	50	1 +	50	0	-	-	0
11	ONB	Hyams 2	95	3 +	285	95	2 +	190	0
12	ONB	Hyams 2	100	3 +	300	100	3 +	300	0
13	ONB	Hyams 2	98	3 +	294	80	2 +	160	0
14	ONB	Hyams 2	100	3 +	300	0	-	-	0
15	ONB	Hyams 3	0	-	-	1	2 +	2	0
16	ONB	Hyams 3	90	2 +	180	0	-	-	0
17	ONB	Hyams 3	95	3 +	285	0	-	-	0
18	ONB	Hyams 3	90	3 +	270	0	-	-	0
19	ONB	Hyams 3	50	2 +	100	0	-	-	0
20	ONB	Hyams 3	85	2 +	170	5	3 +	15	0
21	ONB	Hyams 3	100	3 +	300	90	2 +	180	0
22	ONB	Hyams 3	100	3 +	300	25	3	75	0
23	ONB	Hyams 4	25	1 +	25	0	-	-	0
24	ONB	Hyams 4	0	-	-	0	-	-	0
MTS ONBs									
25	LN MTS ONB	Hyams 2	100	3 +	300	0	-	-	0
26	LN MTS ONB	Hyams 4	-	0	-	0	-	-	0
EP NENs									
27	NEC	SC	0	-	-	0	-	-	0
28	NEC	SC	0	-	-	0	-	-	0
29	NEC	SC	0	-	-	0	-	-	0
30	NEC	LC	0	-	-	0	-	-	0
31	NEC	LC	0	-	-	0	-	-	0
32	MiNEN	ITAC G3	100	3 +	-	0	-	-	100
		NEC	0	-	-	0	-	-	0
Others									
33	AMPH	G3	100	3 +	-	0	-	-	100

*ONB* olfactory neuroblastoma, *MTS* metastatic, *LN* lymph node, *EP NENs* epithelial neuroendocrine neoplasms, *NEC* neuroendocrine carcinoma, *SC* small cell suptype, *LC* large cell subtype, *ITAC* intestinal type adenocarcinoma, *AMPH* amphicrine carcinoma

**Fig. 3:**
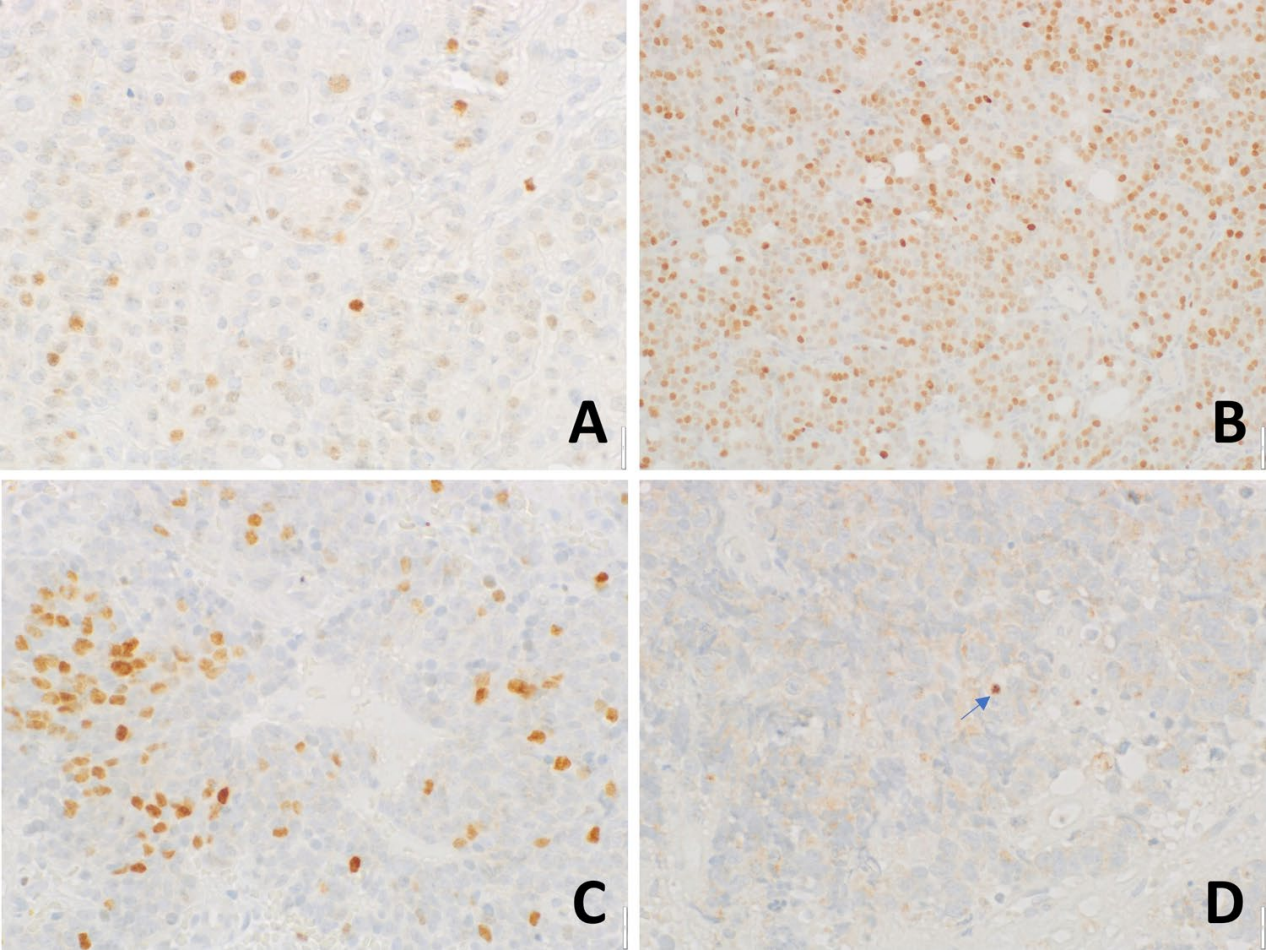
GATA 3 immunoreactivity in ONBs. Hyams grade 1 (**A**), grade 2 (**B**), and grade 3 (**C**) ONBs showed variable immunostain, whereas Hyams’ grade 4 ONBs were consistently negative (**D**)

GATA3 immunostain was heterogeneously present in ONBs: 12 out of 26 cases (46%) stained positive, including 2/2 (100%) Hyams’ grade 1, 6/13 (37,5%) Hyams’ grade 2, and 4/8 (50%) Hyams’ grade 3 ONBs (Fig. 3). Hyams’ grade 4 ONBs, all NENs, and the amphicrine carcinoma were totally negative, as well as the two metastatic ONBs. However, five out of the 12 positive cases (namely, 1 grade 1, 3 grade 2, and 1 grade 3 ONBs) showed only scattered positive nuclei, accounting for less than 5% of the total neoplastic cells. Conversely, 4 cases (3 grade 2 and 1 grade 3 ONBs) were intensely and diffusely positive for GATA3, showing more than 80% of neoplastic nuclei with a moderate or intense positivity (Table 1). Sustentacular cells were negative for GATA3, as well as for SATB2. No direct correlation between SATB2 and GATA3 immunoreactivity in neoplastic cells was found, in terms either of the extent or the intensity of the stain.

**Fig. 4:**
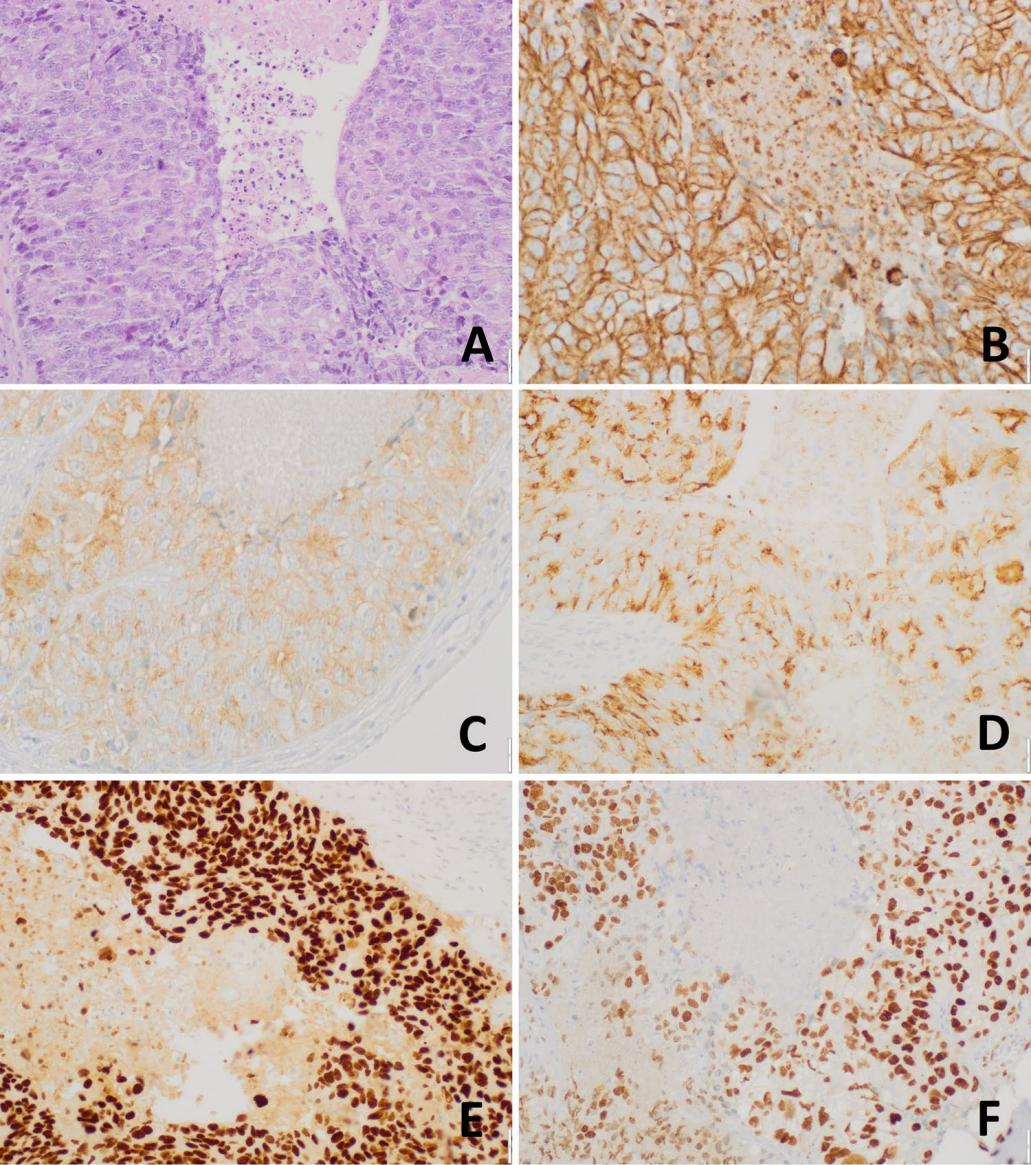
Sinonasal amphicrine carcinoma showing coexistence of intestinal-type and neuroendocrine differentiation. This poorly differentiated sinonasal neoplasms showed large irregular nodules with central dirty necrosis composed of atypical epithelial cells with vesicular nuclei and abundant amphophilic cytoplasm (**A**). CK20 (**B**), synaptophysin (**C**), and chromogranin A (**D**) were coexpressed in the same neoplastic cells, which were also positive for CDX2 (**E**) and SATB2 (**F**)

CDX2 was negative in the totality of ONBs and NECs. As expected, the ITAC component of the MiNEN was positive. In addition, we found an intense and diffuse nuclear immunoreactivity for CDX2 in all neoplastic cells of amphicrine carcinoma, which expressed both general neuroendocrine markers and distal intestine differentiation markers (Fig. 4).

## Discussion

This is the first study reporting the immunohistochemical expression profile of SATB2, GATA3, and CDX2 in olfactory neuroblastoma (ONB). Our results demonstrate that SATB2 is consistently expressed in Hyams’ grades 1, 2, and 3 ONBs, whereas grade 4 ONBs show negative or focal and faint immunostaining for this transcription factor. Neuroendocrine carcinomas (NECs) of the nasal cavity, both of small cell and large cell subtypes, did not show any immunostaining for SATB2, including the NEC component of the MiNEN, where the intestinal-type adenocarcinoma (ITAC) component was, as expected, intensely positive. Regarding GATA3, this marker was heterogeneously and unpredictably expressed in Hyams’ grades 1–3 ONBs, whereas grade 4 ONBs, NENs, and the amphicrine carcinoma were completely negative. CDX2 was only expressed in the amphicrine carcinoma and, as expected, in the ITAC component of the MiNEN.

The expression of SATB2 in ONBs is difficult to be explained. In fact, these neoplasms are thought to originate from neuroepithelial (neuroectodermal) olfactory cells that are normally found in the upper part of the nasal cavity, in which SATB2 has not been found to be physiologically expressed. Indeed, although a low mRNA expression has been detected in the olfactory bulb [20, 21], SATB2 has not been found to be involved in the regulation of the olfactory system, in contrast to its proposed role in taste perception [22]. However, SATB2 is involved in craniofacial and brain development during embryogenesis, and it is conceivable that cranial neoplasms may aberrantly re-express it [23]. Interestingly, in our series, SATB2 expression seems to be a feature of only Hyams’ grades 1 to 3 ONBs, with an apparent gradient of intensity in grades 1 and 2 tumors, which lose SATB2 expression in the most differentiated areas of neoplastic nests, where Homer-Wright rosettes are present. In contrast to grades 1–3 neoplasms, Hyams’ grade 4 ONBs are inconsistently positive. The extensive diagnostic immunohistochemical workup performed to exclude the diagnosis of other morphologically similar high-grade sinonasal neoplasms makes unlikely that the negativity or faint positivity of Hyams’ grade 4 ONBs may be related to intrinsic differences in nature, other than grade, of these tumors from the other analyzed ONBs. This result, together with the distinctive high aggressiveness of grade 4 ONBs [6, 24], further supports their separation from low-grade ONBs and suggests that they are biologically different from grade 3 ONBs, although some Authors have proposed the grouping of grade 3 and grade 4 in a common “high-grade ONB” category [24, 25]. In fact, grade 4 ONBs have a clinical behavior that is more similar to other high grade neoplasms of the nasal cavity, among which neuroendocrine carcinoma (NEC) represents an important differential diagnosis [2]. The existence of molecular and genetic heterogeneity of ONBs has been suggested by recent studies [6, 26] and further endeavors should be devoted to identifying additional markers and signatures able to integrate Hyams grading system in prognostic stratification of ONBs, which may offer new tailored approaches for the treatment and follow-up of such patients [27]. From a diagnostic point of view, NECs of our series were completely negative for SATB2, confirming the inconstant expression of this transcription factor in neuroendocrine neoplasms [12] and making SATB2 not useful as a differential marker between grade 4 ONBs and NEC.

Worth to be noted, the amphicrine carcinoma included in our series was intensely positive for SATB2 in the totality of neoplastic cells. This finding is probably related to the ITAC differentiation in all cells and further supports the separation of amphicrine carcinomas from neuroendocrine neoplasms [3].

GATA3 expression in ONBs expands the spectrum of GATA3-positive neoplasms, which encompasses both epithelial and non-epithelial proliferations, including neuroectoderm-derived neoplasms, such as neuroblastoma, ganglioneuroblastoma, and ganglioneuroma, as well as pheochromocytoma and paraganglioma [28, 29], these latter considered as non-epithelial NENs [19]. Interestingly, the expression pattern of GATA 3 in ONBs differs from that of neuroblastoma and non-epithelial NENs, which are both intensely and diffusely positive in the majority of cases, whereas only few ONBs in our series showed such a convincing immunoreactivity. Although its inconstant expression in ONBs excludes GATA3 as a useful marker in diagnostic practice, the pathologist should be aware of the possibility of a strong and diffuse expression in these neoplasms when discussing the differential diagnosis of a head and neck neoplasms or the metastatic localization of an unknown primary. Besides metastatic localization of GATA3-positive tumors in the nasal cavity, GATA3 expression may be seen in primary sinonasal neuroendocrine neoplasms, like ectopic pituitary neuroendocrine tumors/adenomas (PitNETs) [30] and paraganglioma [28, 29]. In the case of GATA3-positive ectopic PitNET, additional pituitary-specific transcription factors, including Pit1, TPIT, and SF1, as well as pituitary hormones, help in establishing the correct diagnosis. Moreover, among neuroendocrine marker-positive and cytokeratin-negative sinonasal neoplasms, the expression of tyrosine hydroxylase favors paraganglioma versus ONB. It should be noted that also non-neuroendocrine neoplasms of the nasal cavity, namely salivary type carcinoma, may be GATA3-positive [28, 31]. Thus, the results of our study strengthen, once again, the need for practicing pathologist of knowing the features of each biomarker, including their expression profile in different entities. In fact, as in other fields of pathology, the differential diagnosis of morphologically challenging head and neck neoplasms should be always approached using a carefully selected panel of immunohistochemical markers, oriented to distinguish relevant entities. This is particularly true for neoplasms located in the paranasal sinuses and nasal cavity, which are also diagnosed on small and artifactual biopsy samples, particularly in centers that perform transnasal endoscopic surgery.

The third analyzed transcription factor was CDX2, which was not detected either in ONBs or NECs, confirming its specificity for intestinal-type differentiation, as it was positive in the ITAC component of the MiNEN and in the amphicrine carcinoma, where it paralleled SATB2 expression.

In conclusion, our study identifies, for the first time, SATB2 expression as a feature of Hyams grades 1–3 ONBs, which may also stain positive for GATA3. Interestingly, Hyams’ grade 4 ONBs, similarly to sinonasal NECs, showed a faint or, most frequently, absent expression of both markers. Our results expand the spectrum of SATB2 and GATA3-positive neoplasms, with relevant practical diagnostic correlates, and suggest that Hyams’ grade 4 ONBs are not only clinically but also biologically different from lower grade ONBs.
